# Geropsychiatric Care of Older Adults: Substance Use, Delirium and Depression

**DOI:** 10.1093/geroni/igaf122.1561

**Published:** 2025-12-31

## Abstract

In this session the participants will gain insights into research addressing substance use, delirium, depression and suicide in older adults

